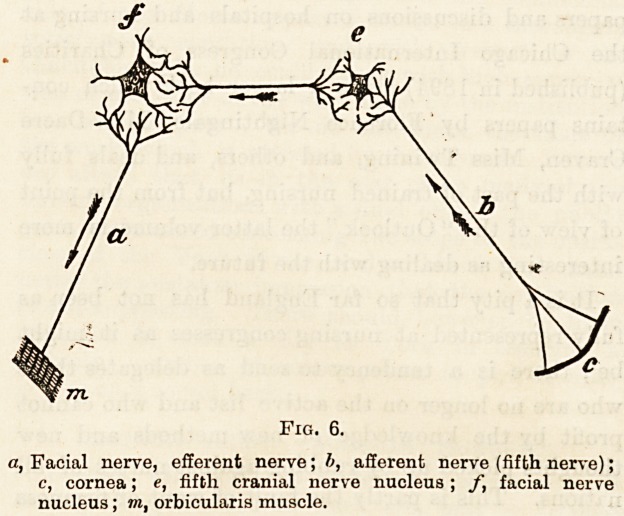# The Hospital. Nursing Section

**Published:** 1903-03-07

**Authors:** 


					The Hospital.
"Ruwtafl Section. X
Contributions for this Section of "The Hospital" should be addressed to the Editor, "Thb Hospitai"
Nursing Section, 28 & 29 Southampton Street, Strand, London, W.O.
NO. 858.?VOL. XXXIII. SATURDAY, MARCH 7, 1903.
IRotes on IRewg from tbe IRursfng TKHorlO.
ROYAL national pension fund for nurses.
The sixteenth annual general meeting of the
Royal National Pension Fund for Nurses will be
'held on Thursday, March 12th, at River Plate House,
l'insbury Circus, at 4 p.m., when the report of the
council, with the statement of accounts, will be
presented. The most striking figures of the report
?were given in our issue of January 3. But it may
be added that, owing to ill-health, Miss Gordon,
late matron of St. Thomas's Hospital, is unfortunately
unable to continue as a representative of the policy-
holders. The council take the opportunity of ex-
pressing to her their sympathy and their sincere
'thanks for the work which she has done for the
Pund since 1891. In Miss Gordon's place the name
?f Miss Florence Smedley, matron of St. George's
'Hospital, will be proposed.
NURSES' HOME AT THE MIDDLESEX HOSPITAL.
The nurses' new quarters at the Middlesex Hos-
pital are nearing completion, and will shortly be in
occupation. They consist of a large dining-room,
sitting-room, and about fifty bedrooms. The dining-
room is wainscotted in light oak, with soft green
colouring on the walls above, and the Teale fireplace
green tiled to match. The colouring of the sitting-
room is pale terra-cotta and white, and the windows
of both, which look into the courtyard, are of small
square panes, with cathedral glass in the lower sash.
The bedrooms on the same side are very sunny and
bright, and have most inviting window seats and
?artistic square-paned windows. Those on the other
side have windows of ground glass, being somewhat
?overlooked by the street. It was stated by Lord
?Sandhurst at the annual meeting last week that the
provision of the new home would enable the com-
mittee to very considerably augment the nursing staff,
though to what extent it has not yet been decided.
The increase in numbers would enable certain con-
cessions to be made, and the nurses would thus have
more leisure than was possible under existing
circumstances. " We have long wished," Lord Sand-
hurst concluded, " to add to the staff, but have not
had space for them." The question of hours, off duty
time and opportunities for recreation and other con-
cessions to nurses are in the hands of the Nursing
Committee, and with the increase of the staff a
considerably improved state of things is looked for.
KEIGHLEY GUARDIANS AND WORKHOUSE
NURSING.
The Keighley Board of Guardians deserve credit
for the care and attention which the memorandum
they have issued shows they have given to the report
of the departmental committee on nursing in Work-
houses, as it affects institutions having no resident
medical officer. There are a hundred beds at the
Keighley Union Infirmary, with a superintendent
nurse, three trained nurses, and six probationers.
The training of the probationers extends over three
years, and the medical officer, as well as the superin-
tendent nurie, gives lectures. He also affords
systematic clinical instruction. The probationers
are examined in theory and practice at the middle
and end of the term by the medical officer of some
large workhouse, and the guardians afford them
pecuniary assistance on their examination for the
certificate of the London Obstetrical Society. There
is an operation room, and major and minor surgical
operations are performed in the presence of the
March 7, 1903. THE HOSPITAL. Nursing Section. 311
probationers. The infirmary has been a training school
for ten years. These are the grounds upon which it
is urged that even without a resident medical officer,
the infirmary should be considered by the Local
Government Board to have a proper system and
opportunity of training nurses adequately. The
Iveighley Guardians suggest " that instead of regulat-
ing the position of nurses solely by their place of
training, all probationers of three years' training
should undergo the same examination, such examina-
tion being satisfactory to the Local Government
Board, and after passing the examination should
receive the same certificate." The certificate they
propose, should specify the place of training, and by
this method, they contend, the best nurses, wherever
?originally trained, would have the opportunity of
rising in their profession, the number of training
schools would be largely increased, and there would
be no unnecessary check placed upon the training of
probationers.
EAST LONDON NURSING SOCIETY.
The annual meeting of the East London Nursing
Society will be held at the Mansion House on
Tuesday afternoon next, the Lord Mayor in the
chair, and among the speakers will be the Bishop
of Barking, the Chief Rabbi, and Sir Dyce Duck-
worth. The report of the committee for the year
1902 shows that upwards of 4,000 cases were nursed,
but that the state of the funds is not as satisfactory
as it was last year, the expenditure having exceeded
the income to the extent of nearly ?150. The usual
interesting tables, with the proportions of men,
women, and children nursed, the occupations of
heads of families, how most of the cases were sup-
ported, and the illnesses nursed, are given. Those
^Tho desire to know precisely the nature of the
excellent work which is being done among the masses
in the East End by the trained nurses in 27 dis-
tricts, and are not able to go to the Mansion House
??n Tuesday should send for a copy of the report to
the Secretary of the Society, 43 Rutland Street,
?New Road, Commercial Road East, E.
the complaint of the workhouse
MATRONS.
"W"e are not surprised that the report of the
Departmental Committee has excited the displeasure
of workhouse matrons, whose position will certainly
be materially altered if the recommendations of the
committee are carried into effect. On one point
there can scarcely be room for disagreement. Exist-
ing matrons should, we think, in any case be allowed
to keep the authority which was given them when
they were appointed. Nor if a matron be a fully-
trained nurse is there any necessity for reducing her
position. The essential thing is, that the chief
female officer of a workhouse should be a fully-
trained nurse, whether she be called matron or
superintendent, and we readily admit that this
salutary reform cannot, without unnecessary harsh-
ness to individuals, be enforced all at once.
THEATRICAL PERFORMANCES AND DISTRICT
NURSING ASSOCIATIONS.
The Rev. T. W. Thomas, vicar of St. Barnabas,
Cambridge, has publicly protested against the Cam-
bridge District Nursing Institution swelling its funds
by receiving the proceeds of a theatrical performance.
We are glad to observe from a report of the annual
meeting of the institution that the benefits it confers
upon the town were warmly recognised by the Vice-
Chancellor and others of the clergy, one of whom
said it was second in importance only to the hospital.
If the strong appeal which was made to the colleges to
subscribe in their corporate capacity to an organisa-
tion whose claims upon them are at least as great as
those of the poor in the East End of London, the
institution would, doubless, be quite independent
of theatrical performances by amateurs. But the
principle enunciated in the protest of Mr. Thomas
is to be deprecated on general grounds. At Clay Cross
a dramatic performance has just been held in aid of
the fund of the local Nursing Association and yielded
some i?50, a sum which was urgently required. The
programme on the occasion consisted of a representa-
tion of "Our Bitterest Foe," an incident in the
Franco-Prussian war of 1870, and "Still Waters
Run Deep." What possible objection can there be
to either of these pieces'/ While we strongly hold
that the best, and indeed the only satisfactory, way
of carrying on a District Nursing Association is to
obtain a sufficient income by means of regular con-
tributions, if this cannot be done extraordinary
efforts on the part of the charitably disposed are
naturally and properly welcomed. We utterly
fail to see that there is anything objectionable in
such an excellent institution as the Cambridge
Association accepting the help proffered by kindly
townspeople through the medium of an entertain-
ment which is only considered harmful by persons
who seem to imagine that it is a Christian duty to
be narrow-minded.
A REMINISCENCE OF RUSKIN.
An old friend of John Ruskin tells an amusing
story of that famous man in Household Words.
After he had been seriously ill of brain fever, Ruskin
was the victim of some strange hallucinations, one of
which was that every time J ackson,his valet, came into
his room he thought " it was the devil that had come
for him." As a matter of fact, the man was inde-
fatigable in his efforts to alleviate his pain and soothe
him. Sir William Gull, who was attending at the
time, was astonished to find his patient get worse
instead of better, and one day Ruskin told him of this
disturbing element, and asked him " if he could find
a pretty female nurse to come and take charge of him."
Sir William?who probably saw the advisability of
humouring a sick man's fancy?said that he could,
and a nurse of this description was got, Ruskin sub-
sequently telling the author of the reminiscence that
" the effect of that pretty face and graceful figure
was a greater restorative than all Sir William's medi-
cine." At all events, when the doctor called again
he was amazed to find Ruskin almost recovered. We
are afraid that if the nurse had been only pretty and
not well-trained and tactful, this desirable result
would not have been achieved.
THE WORKING CLASSES AND TRAINED NURSES.
At a largely attended meeting consisting almost
exclusively of working men, the rules of the new
nursing organisation at Bishop Auckland were con-
sidered and passed with slight alterations. The
second rule is to the effect that the object of the
312 Nursing Section. THE HOSPITAL. March 7, 1903.
association is to provide trained nurses for the sick
poor and industrial classes in their own homes.
Subscribers of a minimum of 2s. per year are to be
able to attend and vote at the annual and special
meetings. It was strongly insisted by some of the
speakers that the first object in view must be " to
nurse the sick poor." A gratifying feature in con-
nection with this movement is the determination of
the working classes not to be content with half-trained
nurses. There are indeed many indications that
wherever the working men take a practical interest
in nursing matters, they are shrewd enough to recog-
nise the importance of putting the work into the
hands of the persons who can do it in the best
manner.
NORTHAMPTON TRAINED TOWN AND COUNTRY
NURSES.
One result of the formation of the Northampton-
shire County Nursing Association, which, in spite of
warnings from many quarters, has been started on the
weak lines deprecated by Lady Knightley, will be the
danger, owing to the similarity of name, of its nurses
being mistaken for those on the staff of the North-
ampton Town and County Nursing Institution. This
institution, though some think it is handicapped by
having both a district and a private branch, employs
only fully-trained nurses, whose excellent work re-
ceived no more than the recognition which it merits at
the annual meeting of governors and subscribers. Last
year the district nurses attached to the institution
paid no fewer than 13,766 visits to the houses of
poor people ; 71 of whom were in receipt of parish
relief. The Northampton Board of Guardians
cannot therefore affirm that they do not receive an
adequate return for the 40 guineas a year which they
subscribe to the association. During the year several
of the nurses were engaged in nursing patients
suffering from small-pox. A striking proof of the
value of house-to-house collections is given in the
annual report. Mrs. Hickson, who took upon her-
self the superintendence of this arduous task, was
able to hand over to the fund the handsome amount
Of ?160.
%
THE GLASGOW " CO-OPERATION."
Apparently the nurses belonging to the Glasgow
and West of Scotland Co-operation of Trained
Nurses prefer to be servants rather than independent
members of the organisation. At all events, at the
meeting from which Mr. Bannatyne was excluded,
and possibly also other persons with the right of
admission, the new constitution was submitted and
approved. If, as there is no reason to doubt, the
proceedings hold good in law, the power of the
committee is now absolute and members who are
not prepared to submit to their dictation are likely
to have a rough time of it. However, people who
have neither the pluck nor the energy to try and help
themselves when others endeavour to assist them
must always expect to suffer in consequence. It
seems as if Nursing Co-operations on both sides of
the Tweed were doomed to fall into the hands of
tyrannical lay committees.
YORK HOME FOR TRAINED NURSES.
At the annual meeting of the governors, sub-
scribers, and friends of the York Home for Trained
Nurses, Dr. Ramsay read the annual report, which
showed that the number of cases attended was 1,103,
645 of which were treated free of all charge, and
458 for payment. The total income for 1902 was-
?3,127, or ?60 in excess of 1901 ; but the total ex-
penditure in excess of the same period was ?107.
The serious point is that with regard to the fund for
nursing and relief of the sick poor there is a deficit
of ?157. Thus, for the last two years the debt has
been increasing at the rate of more than ?100 a
year, and as the report adds : "it is evident that
unless this adverse condition of things can be re-
dressed the work must inevitably suffer." Dr;
Swanson, in seconding the motion for the adoption'
of the report, which was moved by the Dean of
York, said that what he might call the commercial'
side of the Home appeared to be in a thoroughly
satisfactory condition, and with respect to the
charitable side, he could speak with high apprecia-
tion of the value of the work done by the institution
for the sick poor. We do not doubt the value o?
the work, which is attested by the figures. But we
are afraid that, as in the case of so many of the
nursing organisations with a dual object, the public
got a little mixed concerning the scope of the opera-
tions of the York Home, and withheld their hand
under the mistaken impression that the institution
is self-supporting. There should, however, be little
difficulty in such a city as York in securing a
sufficient number of subscriptions to put it alto-
gether on a sound basis.
CHURCH ARMY "MISSION NURSES."
Women who wish to undertake mission work
among the poor, rescue work, etc., may be trained-
under Miss Carlile, head of the Women's Training
Home, 61 Bryanston Street, London, W. The
women thus trained are usually Bible women with-
some knowledge of nursing, which is useful in their
religious work. Their experience of sickness is
gained by daily visits to the Kensington In-
firmary, where, by arrangement with the authorities,,
they are allowed to assist in preparing patients for
the doctors. A lady doctor gives a course of lectures
on nursing, and the First Aid Course of the St.
John Ambulance Association is taken. There are
also weekly attendances at the Church Army Dis-
pensary for Women and Children, 39 Homer
Street, W. These mission women are not allowed
to undertake infectious cases or night nursing without
special permission, as it interferes with their primary
duties, which are evangelistic. Some of them are
drawn from the ranks of elementary school teachers,
shop assistants, mill hands, and domestic servants,
while a few have had hospital training. More thara
200 are employed, and there are vacancies for many
more. The training is free.
SHORT ITEMS.
The s.s. Assaye, which reached Southampton from
India on Friday last, had on board Sisters Hayes,
Lingard, and Goldsmith, of the Indian Nursing
Service ; and the s.s. Sardinia, which arrived on
Saturday from South Africa, Sister A. P. Carruthers,
of the Army Nursing Service Reserve.?In
People's Journal recent literary competition, " How
to Become and Succeed as a Nurse," theprHe of two
guineas has been won by Nurse Anna Sinclair
Quoys, Holm, Orkney Islands.
March 7,1903. THE HOSPITAL. Nursing Section. 313
Che muraitifl ?utloofe.
" From magnanimity, all fear above;
From nobler recompense, above applause ;
Which owes to mm's short outlook all its charm."
CONGRESS REPORTS.
No more interesting volume to nurses has ever
probably been published than the " Transactions of
the Third International Congress of Nurses." The
papers and discussions on hospitals and nursing at
the Chicago International Congress of Charities
(published in 1894) make a larger book which con-
tains papers by Florence Nightingale, Mrs. Dacre
Craven, Miss Twining, and others, and deals fully
"with the past of trained nursing, but from the point
of view of the " Outlook " the latter volume is more
interesting as dealing with the future.
It is a pity that so far England has not been as
fully represented at nursing congresses as it might
be; there is a tendency to send as delegates those
"who are no longer on the active list and who cannot
profit by the knowledge of new methods and new
thoughts picked up by rubbing against nurses of all
nations. This is partly the fault of want of firmness
amongst English nurses, and partly the fault of
hesitating conservatism on the part of nursing
authorities. It will ever be remembered to the
credit of Miss Amy Hughes that she resigned the
important position of superintendent of the Nurses'
Co-operation because leave to attend the last
Congress was refused her by the then antiquated
committee. One would rather have thought that
the Co-operation would have applauded Miss Hughes'
public spirit, appointed her a delegate, and paid all
her expenses. Perhaps we shall see some such
action taken in the future, now that the nurses are
themselves resolved to control their own.
Amongst the subjects discussed at the Congress
were Hospital Administration, Women on Hospital
Boards, Co-operation amongst Nurses, Training of
Nurses, Hourly Nursing, School Nursing and Nursing
Time of War. The last subject naturally loomed
large, for the American nurses had Cuba to look back
on and the English nurses were in the throes of the
trouble in South Africa. Much valuable experience
was to be gathered had the Army Nursing Service
only sent a delegate from England, but it had not.
However, the Indian Nursing Service sent Miss
Arkle, and it is probable that at future congresses
there will be better representation of the English
Government and other authorities. We would
remind nurses that the next Congress will be held at
Berlin in 1904, and therefore the expense of sending
delegates will not be so great, though the foreign
language will probably be a difficulty. It is not too-
early for important nursing organisations to consider
?whether they wish to be represented or not. Tn
some matters Germany is behind us in nursing, in
few it is ahead of us; on the whole there is a lack of
uniformity, a want of some common standard, that
is regrettable. And yet there are certain German
surgeons who have taken the utmost trouble to train
special nurses, and there are clinics that in.
their fittings and appliances are an example for all.
nations. That the best that is to be seen will be
available for the Congress delegates is proved by the
excellent committee now being formed at Berlin to
make the arrangements.
In considering whether to send a delegate, and
who to send as a delegate, it is well to remember the
possibilities of combination. At the Buffalo Con-
gress some 13 different societies were represented by
Miss Hughes and Miss Wood. This was a forward
step, but however able those two ladies are, they were
neither, for instance, experts in the latest asylum
methods, and the remarks on Mrs. Chapman's paper
give a wrong view and are inaccurate in detail. It
was a pity to be able to send only two delegates for
so many branches of nursing. But all the same there-
is no reason why several London hospitals should not
combine to send a delegate to Berlin, or several
district nursing associations should not agree on one
representative ; or all the co operations of nurses in
London could send one representative. If there is
to be combined representation let it be amongst
similar institutions.
And there are certain personal qualifications that,
are desirable in every delegate, and here the senior
must be prepared, perhaps, to make way for the
junior. To begin with, the delegate should be
someone in active work, and in direct touch with
English methods of the day; then she should have
the inquiring mind, the energetic body, and the
agreeable manner, in order that she may learn all
that there is to learn, and be taken into the friendly
confidence of all those she meets. The larger her
knowledge of languages, and of the courtesies so
common on the continent and so rare in England, the
better ; and certainly, for the Berlin Congress, the
power to speak German should be a sine qud non.
Then, finally, she should be capable not only of
attaining the knowledge, but of passing it on to
those who sent her : she should have a clear brain,
and be able in a speech to relate all such experiences
as are of value. Then a meeting can be called on
her return of those who sent her, and they can learn
from her lips what they were not all able to enjoy
personally.
In this way it would be possible not only for
England to gain something from the Congress, but
also to hold honourably the position amongst the.
nations that was founded for her in nursing by
Florence Nightingale.
314 Nursing Section. THE HOSPITAL. March" 7, 1903.
Hectares on ?pbtbalmic IRurslng.
By A. S. Cobbledick, M.D., B.S.Loncl., Senior Clinical Assistant Royal Eye Hospital, late House-Surgeon and
Registrar, Royal Eye Hospital.
LECTURE Y.?PHYSIOLOGY.
The Protective Mechanisms of the Eyes?The
Nutrition of the Eye?Accommodation.
Considering the importance of vision to the human
economy, it may at first sight appear that the provisions
made for its safety are scarcely adequate. Experience shows
that?when we exclude hazardous occupations and certain
accidents?the bony orbit, the eyelids, and a certain reflex to
be hereafter described, form a most efficient protective
mechanism for the eyeball. The value of the bony orbital
margin has already been indicated; it is very exceptional for
blows on the eye with the fist, a very common form of
injury, to cause damage to the eyeball; at most a black eye
?i.e., effusion of blood into the loose tissue of the eyelids?or
fracture of the lachrymal bone results. The eyelids form a
strong curtain which can only be penetrated by considerable
force, on account of the tough fibrous and cartilaginous
layers of which it is composed. The opening and closing of
the lids may be performed at will, but it must not be for-
gotten that " blinking " takes place normally twice or thrice
a minute in an involuntary manner.
The mechanism of closing the lids is performed by the
orbicularis muscle, which is composed of circular strands of
fibres beneath the skin of the eyelids; this muscle is
governed by the seventh'cranial or facial nerve. In cases of
paralysis of, or injury to, this nerve, the lids are open,
blinking does not take place, and by no effort of the will can
they be closed; the consequence is that the front of the
eyeball becomes dry and an easy prey to small irritating
particles.
The lids are mainly opened by the levator palpebrse
superioris, the distribution of which has been studied.
Observe that when the eyeball is raised by looking upwards
the upper lid is also raised; this is due to tendinous
communication between the superior rectus muscle and the
levator palpebrse superioris. The levator palpebral superioris
is supplied by a branch of the third cranial nerve, so that
when this nerve is paralysed there is dropping of the upper
lid. The lower lid is closed by the orbicularis muscle, and
retracted by a band of muscle passing from the inferior
rectus muscle to the lower lid. " Blinking" is a very good
?example of a reflex action. The machinery necessary for
such an action is threefold:?
1. The afferent nerve, which conveys the sensory impulse to
2. The nerve centre.
3. The efferent nerve, which conveys the impulse from the
?centre to the periphery, so producing a muscular contraction.
Applying these facts to the " blinking" reflex, we find
that the necessary stimulation is caused by contact of the
air and minute foreign bodies with the sensitive cornea and
conjunctiva supplied by the fifth cranial sensory nerve; the
impulses set up by this stimulation are carried to the nerve
centre?the facial nerve nucleus?in the brain. The efferent
nerve, which carries the impulse from the centre to the
orbicularis muscle, is the seventh cranial or facial, the result
of this impulse is contraction of that muscle and closure of
the lids, in an endeavour to remove the source of irritation.
The accompanying diagram may assist in making these
points more clear.
The value of this reflex can be readily tested to one's own
satisfaction.
The surface of the cornea and conjunctiva are rendered
moist by secretion from the Meibomian follicles, from some
small mucous gland situated in the fornices of the con-
junctiva, but chiefly by the tears secreted by the lachrymal
gland.
The secretion of tears is another example of a reflex
action : emotion or the presence of a foreign body irritating
the cornea or conjunctiva causes a secretion and flow of
tears.
"Blinking" serves to keep the cornea moist and trans-
parent, for if the cornea is kept uncovered for a few
minutes its surface becomes dry and dim; it also assists
the flow of tears along the canaliculi by pressure on the
lachrymal sac.
The Nutrition of the Eye.?Allusion has previously been
made to the blood-vessels -which supply nutrition to the
different coats of the eyeball. There is, however, another
system which plays an important part in the nutrition of
the eyeball viz. that concerned in the formation and removal
of the aqueous and vitreous humours: it must be carefully
remembered that the eyeball is not a globe containing for
the most part stagnant fluid, but that the contents are more
or less constantly flowing away and reaccumulating, so as
to keep the tension or degree of hardness of the eyeball at
a constant level.
If a puncture is made in the cornea and the aqueous is
allowed to escape, the lens and iris are at once pushed
forward and the anterior chamber appears to be almost
obliterated; if the eye is then bound up for an hour or so,
and at the end of that time it is again examined, the
anterior chamber is found to be full of fluid. The question
is, where does the fluid come from 1 It is no doubt pro-
duced by the ciliary body?whose processes, we have seen,
are arranged around the circumference of the lens?by what
may be regarded as a secretory process. The fluid so formed
at first fills the posterior chamber, and passing forwards
between the iris and lens, fills the anterior chamber.
This circulation is therefore kept up, firstly by the
secretory action of the ciliary body, and secondly by the
removal of the aqueous from the anterior chamber through
the small spaces of Fontana situated at the junction of the
cornea and iris. The spaces of Fontana in turn communi-
cate with the canal of Schlemm (Fig. 1), by which means
the fluid reaches the ophthalmic veins. It is very probable
that the frequent action of the ciliary muscle during accom-
/
Fig. 6.
a, Facial nerve, efferent nerve; b, afferent nerve (fifth nerve);
c, cornea; e, fifth cranial nerve nucleus ; f, facial nerve
nucleus; in, orbicularis muscle.
March 7, 1903. THE HOSPITAL. Nursing Section. 315
modation?i.e. the process whereby the eye adapts itself for
different distances?causing alterate compressions and re-
laxations on the canal of Schlemm, favours the onward flow
of the aqueous by an action similar to that of a suction
pump. The formation of vitreous is probably similar, fluid
passing to and fro through the hyaloid membrane, which
separates the aqueous and vitreous, by a process of osmosis.
Thus it is that the intra-ocular pressure or tension of the
eyeball is fairly constant, only varying slightly with rise and
fall of the general blood pressure. It can be readily under-
stood that if sudden changes took place in the tension of the
eyeball the value of the eye as a visual organ would be much
impaired; the importance of this circulation will be more
apparent when the diseases are dealt with which are caused
by interference with the normal flow of the aqueous and
vitreous.
Accommodation and Movements of the Iris.?In directing
our attention from a distant to a near object we are distinctly
conscious of an effort, but in looking from a near to a distant
object there is a sense of relaxation or passive movement;
this adjustment of the eye for different distances is termed
accommodation. There are limits to this power of accom-
modation if ordinary book print is gradually brought towards
the eyes a point is finally reached where the print becomes
indistinct and cannot be deciphered; the distance of this
point from the eye is termed the near limit of accommoda-
tion. The far limit of accommodation varies in different
individuals according to deviations from normal vision, e.g.
a short-sighted person's far point of accommodation is much
nearer the eyes than a long-sighted person's.
During accommodation three important changes take
place in the eye.
1. The iris contracts, i.e., the pupil becomes smaller.
2. The anterior convexity of the lens is greatly increased.
3. The two eyeballs converge.
The first and third changes can readily be demonstrated.
Ikensington IDtetrict IRurstng Hssoclation,
There was a very large attendance at the annual meeting
of the Kensington District Nursing Association which was
held at Kensington Town Hall on Tuesday. H.R.H. Princess
Louise (Duchess of Argyll), President of the Association,
was present, the Hon. Elspeth Campbell being in attendance.
The chair was taken by Canon Pennefather in the unavoid-
able absence at Manchester of the Dake of Argyll. The
meeting being somewhat late in beginning, the chairman
at once called upon Sir Henry Burdett, K.C.B., to move the
adoption of the report and the re-election of the council and
executive committee.
Sir Henry Burdett commenced his speech by observing
that it gave him great pleasure to say anything in support of
nursing. There were many movements in cities which were
essential to the well-being of the poorer classes, but speak-
ing from a knowledge of the requirements of these classes
and from a pretty wide experience of the worst type of the
dwellings of the poor, he held that there could be no more
beneficent work for the people than that of a district nurse,
who possesses the heart and the soul to carry out her duty
on principle, and always strives to do her best. The work
has been going on in Kensington for some years, and Sir
Henry pointed out that it was carried on at a cost of about
^1,000 a year, and that over a thousand poor families had
been helped in times of sickness in their own humble homes
by the nurses of the Association.
The Value of a Sovereign.
He appealed to his hearers to think for a moment what a
sovereign would do in connection with the labour of such
an organisation as this. Each case cost something like 17s.,
but that small sum expended on a poor family by the minis-
trations of one of the Kensington nurses meant untold
advantages to the household. The nurse brought in light,
and hope, and life. It was a fact, and one which all medical
practitioners recognised, that the introduction of a
nurse into a humble family in a great city meant a
wonderful influence for good. The report showed that
the nurses had paid over 25,000 visits, and each visit
represented a cost of about 8d. The object of the meeting
was that the people of Kensington should have this work
brought to their notice in order that they might have their
hearts and feelings touched, so that more money might be
placed at the disposal of the committee. Was it possible
to expend 17s. in any way in food, in pleasure, or even
in books, which could produce anything like the result
achieved by the attendance of one nurse in a poor
family ? If it were practicable to bring before Kensington
residents at their dinner tables that night an exact repre-
sentation of the incidents in one day's work of one of the
nurses of the Kensington Association, he thought that there
would not be one family who would not hasten to become
associated with the work at least to the extent of ?1 a
year.
"The Angel in the Home."
As to the qualifications of a district nurse, the woman who
engages in such work must have learnt discipline, and must
have an earnest desire to carry out all the instructions of
the doctor. She must love her work and have an
abundance of common sense?a quality difficult to find,
especially in the young. In these days people read so
much about diseases in the newspapers that they often
reduced themselves to the condition of the medical student
on the eve of his examination, when the budding physician
imagines that he has acquired in his own case all human
disease. After perusing accounts of cancer cases people
often felt that they themselves were smitten with the
malady. But in cases where this terrible disease really
existed?in the households of the poor where the husband
or wife has been laid aside by it, unable to minister to their
own needs, and without anyone in the family of sufficient
intelligence to supply those needs?into such a household
the nurse enters, arranges for the children, provides disinfect-
ants, sweetens the unpleasantness attendant upon the disease,
and by her daily visit brings hope to those who are well?
hope that, in spite of the restricted character of their home,
with the nurse's help they may be enabled to keep their dear
one in their midst. It was often most difficult for the poor
to gain admission into the institutions, and they clung very
tenaciously to their homes and desired to remain in them as
long as possible. The district nurse did a splendid work,
and haS been well named " the angel in the home."
The Duty of the Public.
Having dwelt upon the work of the association, mention-
ing how economically it was carried on, and alluding to the
excellent quality of the nurses, Sir Henry proceeded to touch
upon the position the public should adopt as regards the work.
We should thank God, he said, that through the memorial to
our beloved Qaeen Victoria we now had trained nurses work-
ing up and down the land, not only in the cities, but also
in the country villages. Whatever else might be supported,
no one who had any true sympathy with the life of the poor
316 Nursing Section. THE HOSPITAL. March 7, 1903.
KENSINGTON DISTRICT NURSING ASSOCIATION?Continued.
?any realisation of the work of the nurses?could hesitate to
subscribe to such an organisation as the Kensington Nursing
Association. The public owed a duty to the nurses who
have to perform very hard and often unpleasant tasks?so
unpleasant that unless they were women of considerable
character, they could not continue to fulfil them. Those
of the inhabitants of Kensington who had carriages,
or places in the country, or plenty of money to
spend on pleasure should try to lemember that it
would require very little arrangement to take the nurse
for a drive at least once a week, or have her down
in the country for a few days when she needed a little
rest. These nurses were a very valuable asset to the com-
munity amongst whom they toiled. They inculcated habits
of cleanliness amongst the poor and taught sanitation. In a
thousand ways, the work of district nurses, apart from the
direct advantage to patients, had such a beneficent influence
that wherever it was introduced the cry was for more and
more nurses?not merely as nurses, but also as the means
of educating the poor in the sound principles which tend so
much towards happiness in their daily lives at home.
Mrs. Symes Thompson contrasted the way in which people
regarded nurses some years ago and the light in which they
were looked upon now. She also dwelt upon the inestimable
services rendered by the nurses in instructing the poor
about open windows and such essentials.
Sir H. Seymour Kiog, M P., drew an eloquent picture of the
transformation effected by the presence of the nurse in the
wretched home and begged his hearers to individually
promise, besides subscribing themselves, to get at least one
additional subscriber to this excellent charity.
The announcement was then made by the chairman that
the Princess Louise had subscribed ?20 to the funds and the
Duke of Argyll ?10, and the Mayor of Kensington the same
amount.
A vote of thanks was cordially voted to H.R.H. Princess
Louise, and the proceedings terminated.
The most noteworthy event of the past year, according to
the report, was the opening of the branch home at Kenley
Street, Notting Dale. The subscriptions during the year
showed an increase of ?100, and the total receipts covered
the expenditure.
preparation for an a Nominal ?peration in a private Ibouse.
EXAMINATION QUESTIONS FOR NURSES.
rlHE question was as follows:?(1) If you had only 24
hours in which to prepare for an abdominal operation in a
private house, what steps should you take with regard to the
room to be used, and the patient herself ? (2) What pre-
parations should you make to assist the surgeon to the best
of your ability ? It is presumed that he would briDg all
necessary diessings and antiseptic solutions.
The First Prize.
In preparing the room all furniture, except what is abso-
lutely necessary, must be removed.
If the floor is covered by a carpet, it would not be wise to
take it up at such short notice on account of the extra dust
it would make; the better plan would be to cover it all over
with some clean drugget or sheets.
If the boards are bare, or covered with oilcloth, they
should be well scrubbed ; also all ledges of windows, doors',
or anywhere the dust can lodge, should be wiped down with
a damp cloth.
An operating table will be required, and, failing any other,
? substantial kitchen table, or two small tables placed
together, answer the purpose well; whichever is used must
be perfectly clean.
Two or three small tables will be necessary, on which to
stand bowls, dressings, instruments, etc.
The long table must be covered with two or three
thicknesses of blanket, and over this seme kind of mackin-
tosh, the ordinary white American cloth does very well for
the purpose ; the small tables should also be protected with
the same material. Two extra pieces should be provided
to place over the patient above and below the abdomen.
A foot-bath or pail will be necessary to receive fluid or
dirty sponges, also an abundance of basins and dishes for
instruments, dressings, etc.
Two or three hot-water bottles should be secured, and
a fire lighted in the room some time before the operation.
If possible, the patient should have a bath the evening
before, if not she must be thoroughly washed all over,
the abdomen receiving special attention. This must be well
washed with soap and water and the pubes shaved; it should
then be rubbed with turpentine and ether, sponged well
with some antiseptic lotion, and finally covered with a
sterilised compress, either put on as it is or wrung out of
some lotion; this must be protected with gutta-percha tissue
and kept in position by a bandage.
She should be given an aperient the night before and a
soap-and-water enema in the morning, and if unable to
pass urine immediately before the operation, she must be
catheterised.
No solid food should be given for at least five or six hours
before the operation, but a cup of beef-tea, or an egg beaten
up in milk, may be permitted, about four hours before.
Her clothing should consist of a loose, clean night-gown,
and if the legs are protected by woollen stockings they will
aid very considerably in securing additional warmth for the
patient. A clean blanket or rng should be thrown over them
when on the table.
If there are any false teeth, they should be removed
beforehand.
Having secured a fish-kettle or clean saucepan, should
sterilise some towels, and put in carbolic lotion, in readiness
to place over the mackintoshes which cover the patient, and
on which the surgeon will place his instruments.
Two or three will be required to cover the tops of the
smaller tables, on which the dishes will be ready to receive
his instruments, dressings, lotions, etc.
There must be plenty of hot and cold sterilised water, and
a kettle should be kept boiling on the fire.
The steriliser should also be boiling for the instruments
when they arrive.
Plenty of water, a nail-brush, soap, and towels must be
got ready for his hands.
A dish should be in readinesss to receive the anesthetist's
things, and a receiver and towel in case of sickness.
Excelsior.
Second Prize.
(1) First thing I should do would be to try and keep my
patient as quiet and bright as possible, then I should
prepare a room as near my patient's bedroom as possible
" for operation," first to remove carpet or in case of no time, to
tack sheets firmly down to prevent dust rising, and remove
all unnecessary furniture and have a fire burning in
room. For operating table, the kitchen table may be the
best and placed near ?he window. Two small tables or
boxes covered with white table cloth or towels, would
instruments or lotion bowls, one or two pails and a sma*J
bath would be useful, plenty of boiling water, also c0^
sterilised water, a fish kettle with water ready for the
instruments to be boiled, a few sterilised towels, two or
three mackintoshes or a few yards of American cloth.
(2) In trying to assist the surgeon, I should thus prepar?
the patient. First shave abdomen, then bath patient, an A,
able to take castor oil, I should give jiv to jvi, about
March 7, 1903. THE HOSPITAL. Nursing Section. 317
hours before operation, and 12 hours after soap and water
?enema; if the hair is long put it in two plaits (any false
teeth remove them), then wash abdomen well with soap and
water, turpentine, and methylated spirit, and put a compress
of carbolic, 1-80, if skin is not too tender, and as much
nourishment as time would permit, until about five hours
before operation. If patient is not able to take nourishment
owing to persistent vomiting, I should give her sips of hot
Water, or if thirsty rinse out the mouth frequently.
_ The bed should be made up clean to receive patient, a
Pillow for under the knees, and two bricks or wooden boxes
would do for blocks, and a stool will answer the purpose of a
Cage and a few hot-water bottles.?Estelle.
This month's papers are very good. It is very satisfactory
be able to say that the standard reached is good, very
much above those of several preceding competitions. A
?ood deal of sound, practical knowledge has been displayed,
and considerable ingenuity shown in overcoming difficulties.
The papers are naturally not faultless, and the old error of
attempting too much comes to the front. For instance, it
would be almost impossible to take up a carpet, and (as one
?energetic competitor suggests) rub the wall-paper over with
bread crumbs all in 24 hours, when you consider how much
?^se there is to be done.
The Prize Winners.
The matter is good but the manner is bad. Let me beg of
you to devote a little more thought to lucidity of expression
" Excelsior " comes out first among a large number of good
competitors because she recognises the inexpediency of
moviDg the carpet when time does not allow the dust to
settle. She is also particular about the sterilising of the hot
and cold water and does not omit to mention mackintoshes
and sterilised towels to place over the patient above and
below the abdomen. Her account of how to apply the com-
press is most confused, but her meaning doubtless is, that
it may be simply saturated with sterilised hot water, or
with some solution. In the last few lines, too, it is only by
the light of nature that we discern whose hands are to be
benefited! " Estelle " is equally careless in her manner of
writing, though the substance of her answer is good. I
think, however, that a compress soaked in a 1 in 80 solution
of carbolic would be too weak to be of much use.
Honourable Mention.
This is gained by "Therese," "Beginner," "Kathleen,"
and " Kensington." The last named sends such an excellent
paper that she would have received the first prize, but that
she omits to mention the necessity for sterilising all water
used.
Question foe March.
(1) How would you proceed to make up a fire and to keep
it going noiselessly in the room of a patient sensitive to
sound? (2) How would you prevent the light from it
annoying the invalid 1 The Examiner.
?be JBrooftlgn 3nstitute for IRurses anb flursing.
BY A CORRESPONDENT.
The private nursing home, known as the Brooklyn Insti-
tute, is situated in Sydenham, one of the most healthy spots
lQ the neighbourhood of London. It consists of three
0Qses, numbers 154, 156, and 158, Anerley Road, and is
c^ose to the Crystal Palace. Two of the houses are devoted
? the patients, and the third, recently added, is the nurses'
^ome. The rooms are very light, and have large windows
which let in a plentiful supply of fresh air. At the back
are doping gardens with pleasant lawns. About 10 patients
are received for operations, maternity, and medical treat-
*nent> and the terms are from ?3 Bs. to ?10 10s., according
0 the nature of the case and the position of the room
?ccupied, some, of course, being smaller than others. Two
*?oms are reserved specially for surgical cases. The terms
c ude nursing; doctors' fees are of course not included,
lents' friends are received when necessary. One of the
*"ooms at the back of the house has been fitted up during
e past year as an operation-room. The walls are white
?d, and the furniture is by Messrs. Downes, of white iron
J* glass. The plated sterilisers rest on a brass-topped
e The operation-table is of wood, covered with stuffed
erican leather cloth. Before and after every operation
e walls are washed with carbolic.
Covered passages connect the nurses' home with the rest
the institution. All the rooms are simple but comfort-
le J they have a charming sitting-room on the entrance
?0r> looking on the garden ; this is provided with a piano
^nd easy chairs, and is an ideal place in which to rest
?tween cases. The dining-rcom is in the basement of the
?ther house, and is only used for meals. All the rooms
throughout are laid with floorcloth which is frequently
hashed, and rugs which can easily be taken up and shaken,
air of extreme cleanliness pervades the entire three
ouses. The patients have, of course, their own sitting and
diniDg-rooms, and there is a smoking-room for conva-
lescents, opening on to the garden.
The nurses of the private staff must be hospital trained.
They sign an agreement to serve as a nurse in or out of the
Home, for one month, and to continue month by month
subject to one month's notice on either side. Salaries begin
at ?30 for the first year, with board and lodging between
cases and uniform (indoor once a year and outdoor as
required after the first year), and 10 per cent, on earnings.
The second year's salary is ?35, uniform and 10 per cent, on
earnings, and the third year's nurses receive ?40 with the same
additions. In case of illness, salary is paid for one month from
the date of the beginning of the illness, and nurses are cared
for in the Home. An important clause provides that, upon
the termination of the agreement, either by notice or other-
wise, a nurse shall not act as a nurse, either on her own
account or for any other person, company or institution, or
at any place within six miles of this institution for a period
of four years. One month's holiday in the year is allowed.
There is a permanent staff of 20. The uniform is black
relieved by red, and the monogram " B.I." is worn on the
left sleeve.
The superintendent is Mrs. Cameron, late matron of the
Princess Alice Memorial Hospital, Eastbourne, where she
held the position for five years. She began her hospital
training at Edinburgh, and was subsequently at West-
minster Hospital for three years, both in the hospital and
on the private staff; she took the place of the matron
occasionally. Mrs. Cameron insists on private dress being
worn between cases and in all public places of entertain-
ment. She encourages the nurses to take an intelligent
interest in outside affairs, and deplores the tendency of
nurses generally to become "narrow-minded." She is an
ardent advocate of the registration and inspection of all
nursing homes. It is seven years since Mrs. Cameron took
over the management of the Brooklyn Institute.
318 Nursing Section. THE HOSPITAL. March 7, 1903.
j?\>er?t)o&\>'$ ?pinion*
[Correspondence on all subjects is invited, oat we cannot in any
way be responsible for the opinions expressed by our corre-
spondents. No communication can be entertained if the name
and address of the correspondent are not given as a guarantee
of good faith, but not necessarily for publication. All corre-
spondents should write on one side of the paper only.]
ETIQUETTE.
" A Sister " writes : In answer to ?' J. D.'s " inquiries in
Notes and Queries, may I advise her that the only book
which I recommend is that which is known to most
sensible women?in hospital and elsewhere?as guides to
etiquette?commonsense, innate courtesy, and keen observa-
tion? I think the initial outlay will be well worth the
expenditure, and will not prove unduly expensive.
DISTRICT AND PRIVATE NUR3ING IN TORQUAY.
"An Old Londoner" writes from Torquay: Concerning
the Note in last week's Hospital, headed, "District and
Private Nursing at Torquay," it would be a pity for any
nurses to think there is a good opening here for private or
other nursing. There are, I believe, ninety nurses here
working on their own account, and depending on their own
doctors to give them work, and here is a fairly large private
staff belonging to Kent House Medical and Surgical Home.
This has been established about two and a half years and is
used greatly by all Torquay doctors. Nursing has been
very slack in Torquay since September last, some nurses
having no work at all for some time, the town having been
exceptionally healthy and the season beginning late. It
seems to me that it is a pity for fresh nurses to come here,
as I hear that the doctors are still very slack as regards
cases needing nurses.
NURSES AT CONSUMPTION SANATORIA.
" P. B." writes: I do not think the lady superintendent of
Rostrevor Sanatorium quite understands that it was of
tubercular patients we were writing, not typhoid, and may I
point out to Dr. Reinhardt that the celluloid serviette
cylinders are not very satisfactory ; being open at both ends,
the serviette is continually falling out in its transit from the
pantry to the dining-room, and does not always get returned
to its right case. The linen bags he refers to, which are used
at another large sanatorium, are handsomely embroidered,
and not only add to the table decorations but, as they fasten
securely and have the number of the patient's room on, there
is no risk of mistakes being made; they also have the ad-
vantage of being washed and boiled once a week. Celluloid
can also, I believe, be washed in boiling water, but it does
not tend to improve it.
POOR LAW NURSES AND PENSION FUNDS.
" B. H. writes: I was very much interested in Miss
Wilsons article anent "Poor Law Nurses and Pension
I unds, and would like to say that one reason why nurses
do not join the P und is because it is just the same as pur-
chasing an annuity. At least I have always understood so.
A large proportion of nurses have the " saving spirit" and
bank regularly at the Post Office Savings Bank, and some
even invest in other safe concerns where they can get a
little more interest for their money. It is not, in my opinion,
that nurses despise a pension of ?15 a year, but they prefer
saving in such a way that some of their relatives may benefit
at their decease. Of course, for a nurse with no poor rela-
tions?and there are no doubt many such?the pension fund
is a very wise investment. " Every little makes a mickle,"
and ?15 yearly added to ?20 from some other source is not
to be despised.
THE SALARY QUESTION IN THE COLONIES.
"An Occasional Correspondent " writes: It was in no
carping spirit that the article upon the Salaries of Colonial
Nurses in your issue of November 15th was written by one
who is quite ready to acknowledge the willingness of the
Government to right the wrongs that are expressed but
rather from a wish to help those who, as official employes'
cannot help themselves, especially when as affecting so small
a community their case is likely to remain unrepresented.
Accidents will happen in the best regulated Governments,
and I for one should be glad to enter the sterling salary
scheme in that category, unless it undergoes revision and
offers an equivalent of what the contributor under " Nursing
in the Crown Colonies," dated November 22nd, rightly states
was an agreement to receive half salary at 3s. to the dollar
and half at 2s. But from this the circular note is a thing
separate and distinct; in fact, if accepted?and as before
remarked the scheme was to become compulsory after five
years' service?it annuls the existing agreement entirely.
Those who have read these intelligently learn that the
appointment is a probationary one for a period of five years,
but they also learn that such service would ultimately count
towards a pension, and this is a point not to be ignored
when contemplating the future, for nurses who have spent
three or four years in a training school and a like number
abroad realise that home institutions prefer younger blood to
theirs, and that experience of tropical diseases, however
valuable in the East, is not a very marketable commodity in
the land where these, in their acute stages at least, are
practically unencountered and unknown. And, facing these
difficulties, a nurse may feel herself compelled to accept
terms which in effect reduce her present salary by one-fifth.
CIVIL HOSPITALS AND THE A.N.S.R.
"Reserve Sister" writes : May I be allowed a few final
words on one point in my article which has been somewhat
obscured by subsequent letters on the subject ? The question
was not whether private nurses having served in South
Africa could return to their work, nor whether those civil
hospitals which, at the request of her Majesty or of the War
Office, sent certain members of their staff to South Africa
(allowing them to join the A.N.S.R. for the purpose) would
receive them back on their return, but whether it was right
that membership of the A.N S R. should debar a nurse from
hospital appointments, and whether it was either justifiable
or patriotic to refuse to appoint a member of the A.N.S R-
to a vacant post on the grounds of such membership. I
quoted the words of a matron of a London hospital who
said " she would never appoint a member of the A.N.S R , no-
matter who or what she might be, and she should advise all
other matrons to follow her example." The Hon. Sydney
Holland, on behalf of the civil hospitals, appears to excuse
this attitude on the ground of the convenience of the
hospitals. I, as a member of the A.N.S.R, resent it as
injurious and unjust, both to the A.N.S.R. and to its
members.
STATE REGISTRATION.
Miss Helen Todd, matron National Sanatorium, Bourne-
mouth, writes: May I ask you to allow me a short space m
your columns in which to explain once more the attitude of
the promoters of State registration ? The question of their
motive occupied so great a part of " A Hospital Sister's
former communication that I must be forgiven for imagining
that she thought it of importance. I most certainly do.
was also under the impression that the question bad been
long enough before the public for nurses, at all events, to
have understood that what we seek is an Act, on somewhat
the same lines as those in operation in New Zealand an
elsewhere, defining a training school, a curriculum, anC*?
qualified nurse, registering those who comply with the con, '
tions laid down, and, in order to prevent injustice to tn
nurses of the present day. legislating especially for bona
nurses working at the time of the passing of the Act, bo
who do not possess the specified qualifications. I must n
trespass on your courtesy to do more than affirm the eleD^?fc
tary principles of the measure. Common sense shows tn
the details require earnest deliberation before the draftmi& .
the Bill to be laid before Parliament; that the de ^
may there be altered before the Bill becomes law; and ^
principles must rank before details. At present the state ^
affairs is shortly this : any woman after a few months
ing (or indeed no training at all) is at liberty to call her-
March 7, 1903. THE HOSPITAL. Nursing Section. 319
nurse, to be passed off as such on an unsuspecting public
y unscrupulous superintendents of nursing homes or district
ssociations to the danger of the sick and the detriment of
e nursing profession. We believe this can only be remedied
y the State registration of graduates of nursing training
cq?o1s wll0 aione sh0uld be entitled to style themselves
rained nurses," and to recover fees earned under that
atne. The storm raised by the recent suggestion to create
I a*Vn^er*0r Srac^e ?f qualified nurse in workhouse infirmaries,
I nd the protest signed by such a representative body of pro-
I tCrnal an^ men an(^ women (who thus bore testimony
of lu necessity of a thorough training for a nurse) is a sign
i ^mes evidently not observed by a hospital sister. We
re r ^ave graduated in recognised training schools fully
ahse that three years is all too short for the amount of ex-
perience and theoretical knowledge that must be acquired
etore any person is fit to be called a trained nurse. We do
wish to prevent any woman gaining her livelihood, but
e raise a protest against this being done in a fraudulent
anner by her posing as a "trained nurse "when she is
g.ej;ely an " attendant" or " useful worker " (if a " Hospital
er " prefers that term) amongst the sick.
NURSING BY ORDERLIES.
' Late A.N.S." writes: I have hitherto abstained from
lilng part in the discussions in your paper upon this
subject, realising that a fire which is unfed soon dies out
cannot, however, on reading the letter of " Private Power
vpublished in the current issue), refrain from asking, Is
any good purpose served by continuing to publish a corres-
pondence which has become personal and unseemly 1 The
green-eyed monster" which peeps so plainly through
e "ues of the letter will, I think, lead most people to
ppraise its true value, or, I should grieve, lest it should give
outsiders a very mistaken idea of the service. After some
an^T> exPerience in military hospitals both as Regular
a Reserve, I can look back on multiple instances in which
y efforts for my patients would have been in vain but for
y orderlies' help and co-operation ; of course I have some-
nnes had bad ones, just as there may be bad medical
of ClrS 0r nursing sisters, but I am convinced not one
them would have written of me in the spirit mani-
ested by your correspondent, and I beg that you will
, ot judge the corps by his letter. The orderly has had a
ard time in this campaign, long hours and unpleasant
les have often been his lot. I think this has been fully
ecognised by most sisters, many of whom I know, take a
^?a^and active interest in the men working in their wards.
ndoubtedly the position is sometimes difficult for both;
??e has all sorts to deal with, and is often misjudged.
Sometimes, as Faber puts it,
" Dark hearts in flowers where honey lies
Only the poison will find."
but rests with the individuals to strive to maintain by
Mutual respect and forbearance, that good feeling which is
^ssential. Much money and thought is being lavished upon
j ne.w service. Is it not an opportunity for sisters and
orderlies to join forces in working for the common good 1
ursing is one of the noblest of professions, but training is
useless for either man or woman unless the nurse be
animated by a right spirit and worthy motive. As one who
Wl?hes well to the service (in which I include sisters and
^rderlies), I would beg both to refrain for the future from
he very undignified procedure of throwing mud at one
another; the mud soon brushes off, but the stain of it is apt
t0 be more lasting.
Death in ?ur iRanfis.
We have received an intimation of the death from enteric
fever of Miss Jessie Gordon Rattray, at the" District In-
firmary, Ashton-under-Lyne. Miss Rattray had been engaged
at this infirmary for twelve months, and by her quiet and
kindly manner had rendered herself 4very popular among her
fellow workers.
Appointments.
[No charge is made for announcements under this bead, and we are
always glad to receive, and publish, appointments. But it ia
essential that in all cases the school of training should ba
given.]
Banstead Road Schools.?Miss Jennie Burke has been
appointed charge nnrse. She was trained by the Meath
Workhouse Nnrsing Association.
Darlington Fever Hospital.?Miss Katherine Rowby
has been appointed ward sister. She was trained at East
Pitton Hospital, Leith, for three years, where she has since
been sister for four years. She was also sister-in-charge of
the Small-pox Hospital, Edmonton, for one year.
East Parochial Hospital, Dundee.?Miss Annie Love
has been appointed charge nurse. She was trained at Mill
Road Infirmary, Liverpool, and has since been staff nurse at
Brompton Hospital, London.
Eastington Workhouse.?Miss Mary Coffey has been
appointed assistant nurse. She was trained by the Meath
Workhouse Nursing Association at St. Peter's Home, Woking.
Hospital for Infectious Diseases, Woodbridge,
Guildford. ? Miss Elizabeth Annie Walker has been
appointed matron. She was trained at the Middlesex
Hospital, London, and has since been charge nurse at the
Western Hospital, Fulham, night superintendent and
assistant matron at the Hospital Ship, Dartford, and
assistant matron at Long Reach Hospital, Dartford.
Isolation Hospital, Eastry.?Miss Lelia Parry has
been appointed lady superintendent. She was trained at
St. George's Hospital, London, and has joined the Meath
Workhouse Nursing Association.
Staffordshire General Infirmary, Stafford.?Miss
Mary Preston has been appointed matron and superintendent
ot nursing. She was trained for three years at Liverpool
Royal Infirmary, and has since been sister at Worcester In-
firmary and assistant matron at Swansea General Hospital.
TRAVEL NOTES AND QUERIES.
Swiss Holiday (Yungfrau).?It entirely depends on the length
of your stay whether or no you spend more than ?12. Your
journey to Meiringen and [back will be ?5 10s., and you can get
accommodation in June a3 low as 5 francs per day, which would
be ?1 8s. 6d. per week, so you see just how far your money would
go. You must remember tips and excursions, but the latter are
not dear at Meiringen, there is so much to be done on one's feet,
and by the Brunig mountain line. I do not like public personally-
conducted parties, but the gentleman, whose address I sent your
sister, is not doing it for profit, and everything is very comfortable ;
if my memory serves me the cost is ?8 for three weeks, and ?10
for a month. Write again if you cannot decide.
Venice, the Tyrol, and Dolomites (Royal Oak).?Thank
you for your very kind letter. It is always nice to hear from old
correspondents, and especially pleasant to know that my advice
was helpful. The tour you inquire about has nothing to do with
me, but I know it to be delightful, and I thought our readers
might be glad to hear of it. If you will send me a stamped
envelope I will give you the address, as it is private, but I am
afraid it will be no help to you as you must go first to Munich. I
am afraid you are attempting a little too much in your tour. Why
not restrict it either to the Tyrol or the Italian lakes and Venice,
but not try to do all three ? Moreover, I am afraid it is too early
for the Stelvio Pass, etc. . ? ? but I will make inquiries and tell ?
you if you will keep an eye on this column. 1 he Dolomites are
delightful, and Cortina is charming.
320 Nursing Section. THE HOSPITAL. March 7, 1903.
Ccboes from tbe ?utsi&e Morlb.
Movements of Royalty.
The King remains at Buckingham Palace for the present.
The Prince and ?Princess of Wales have become his
guests for a month or so, whilst the repairs now in
progress at Marlborough House are completed, and the
contents of York House?the late residence of the Heir
Apparent?removed and arranged at Marlborough House,
ready for the return of its new owners. The Queen is
coming to town so as to be present at the first Courts
of the season?on March 13th and 20th?after which she
will probably go to Copenhagen to visit her father the
King of Denmark. Meanwhile, she has been remember-
ing those charities in which she is especially interested, and
through the Dowager Duchess of Manchester has forwarded
to the Davos Sanatorium a donation of ?25. In a letter to
the Duchess she says, " I hope my donation may be of some
use to the poor patients; my patronage of the institution
you had long ago."
Queen Victoria and Ladysmith.
IN addition to the dinner in London on Friday, a tea was
given at Exeter on Saturday to soldiers in commemoration
of Ladysmith Day. General Sir Redvers and Lady Audrey
Buller were present, as well as Dr. Herbert Ryle, Bishop-
desigr ate of Winchester. The latter, whilst expressing a hope
that Ladysmith Day would be observed for many years to
come because of the brave deeds which were accomplished
and the undying honour which those who took part in the relief
had earned, said that at the time when attempts were being
made to reach the besieged town he was on a visit to
Windsor Castle. The effect upon Queen Victoria of the
news of the relief was something of which one could only
speak in terms of the greatest reverence and affection, for
her heart beat for the British Army. So great was her
anxiety that the clergy preaching before her were forbidden,
before the tidings came, to make any reference to the war.
Two days after the news of the success attending the last
effort of Sir Redvers Buller arrived, he (Dr. Ryle) was to
preach before her late Majesty, and five minutes before the
service began he received a message from her to the follow-
ing effect: " The Queen particularly desires that there shall
be a reference to what has occurred." A heavy load had
been lifted off her mind by what her soldiers had done.
Lord Methuen and Mrs. Delarey.
In a book just published upon the South African War,
the authoress, the wife of the chivalrous General Delarey,
makes some interesting allusions to Lord Methuen. Mrs.
Delarey, having had all her sheep and cattle taken,
heard that Lord Methuen had requisitioned her last
two horses. So she went to see him. He asked her
courteously what he could do for her. She replied
" For me you cannot do much, but I have still two
horses left, and one of them belonged to my son, who is
dead, and I hope you will not take it away from me." Lord
Methuen gave her his hand, saying, " It shall not be taken
away from you." Later on, Lord Methuen found it neces-
sary to destroy Lichtenburg because General Delarey and
others used to return home at once as soon as the coast was
clear. So the wife and her children began their long
wanderings with a waggon, trekking from place to place
while the war raged around them. Then Mrs. Delarey
heard that Lord Methuen was wounded and a prisoner, and
she called upon him. When he heard she was there Lord
Methuen said she might come in, that he would like to see her.
And when she had entered he begged her to forgive him for
all the annoyance he had caused her, and asked her if she
had suffered much discomfort from all the running away she
had had to do. Then she inquired in turn if his leg hurt him
much, and he said " No, not very much," and she rejoined " If
your leg gets cured so quickly, then you will come and shoot
at us again." Lord Methuen laughed, but he said " No, I am
going away. I will not shoot at you any more."
The New Minister of the City Temple.
The prominent position of minister of the City Templer
which was rendered vacant by the death of that remarkable
man, Dr. Parker, was filled on Monday by the appointment
of the Rav. R. J. Campbell, of Union Church, Brighton.
Mr. Campbell, who has been preaching at the City Temple
for some time on Thursday mornings to crowded congre-
gations, was born in 1867, and has made his way very
rapidly. He was educated at University College, Notting-
ham, and Christ Church, Oxford, where he took honours in
modern history and political science. His first and only
charge was at Brighton, which he has held since 1895.
Mr. Campbell is a native of London, and both his father an<3
grandfather were Nonconformist ministers.
The Disastrous Gales
The violent gales last week and this naturally caused
much damage, not only to property, but to life. At
Holyhead a newly erected iron church was lifted from
its foundations and crashed in a heap, burying alike
pews, pulpit, and floor. At Hetton-le-Hole, Durham, a
Methodist minister and his wife were in bed when a chimney
stack crashed through the roof, burying the occupants of
the room in wreckage. When extricated the minister was
dead, and his wife is not expected to recover. At New-
castle one of the walls of St. Matthew's church was blown
in bodily and two porches were carried away. At Houghton-
le-Spring the roof of a house was blown off and hurled a
hundred yards further up the street. Everywhere telegraph-
poles were thrown down and the wires snapped. This had'
a disastrous effect on the Furness mail train. When it got
on to the Leven Viaduct at Ulverston it became entangled
in the wires and poles, and while waiting in the middle of
the bridge to have the debris cleared away, the force of tbe-
wind overturned ten carriages. Thirty-two persons were-
injured, mostly by broken glass. Those who were extricated
unhurt found the force of the wind so strong that they coultf
not stand upright, and had to crawl on their hands and
knees to get off the bridge. Four people, including two-
children, are missing, and it is feared lest they may have
been blown over into the swollen river beneath. From Brest
comes news of the wreck of the British steamer OttercaPs'
and loss of 30 lives; and on Monday the mail boat between
Dover and Calais very nearly stranded on the Goodwin
Sands. Seamen have been washed overboard, even wheD
the ships have not been wrecked, and the lifeboats have-
been working night and day. At Padstow, as there were-
not enough men present to get out the boat, the eight wives
and daughters of the Trinity pilots managed to do so.
Herr Kubelik s Engagement.
An interesting announcement is made in a German pap^T
of the approaching marriage of Herr Kubelik, the celebrate"
violinist. He is said to be engaged to Counte3s Marianne
Csaky, a daughter of Count Vidor Csaky, a member of tbe
Hungarian Upper House. The lady is a near relative of t?e
Premier of Hungary. They will be a very young couple, W*
Herr Kubelik has not long attained his majority, and &1:r
fiancee, notwithstanding the fact that she is a widow,a
year his junioi. It is reported that Herr Kubelik has been
attached to her for three years, though it is only now ' .
matters have been definitely arranged. The wedding
not take place until next year, and then Kubel'ik, though
will of course frequently travel on tour as at present, wi
live at Vienna.
J
March 7, 1903. THE HOSPITAL. Nursing Section. 321
H Booh ant> its Storv.
THE DAUGHTER OF A RUSSIAN JEW.*
Among the multitude of novels that are published daily,
many of which have.no claim to distinction, it is a decided
relief to come across a book like " The Circle," in which
there is power, vitality, and an intense realisation of both
character and incident. The interest never flags, and the
reader is kept alert from the first page to the close of the
story.
The character of Anna the heroine, the daughter of a
Russian Jew sheltering in London, who lived near the
Docks and kept a store for the reception of curios of rare
value, is a very remarkable one. Her father, old, and entirely
taken up with his books and business, took little notice of
the daughter growing up by his side and of the possibilities
of her nature gathering force by repression amid the narrow
surroundings. The faithful service rendered to her parent
failed to fill the wider horizon towards which she yearned.
The extraordinary strength of her individuality is forcibly
presented. Here is the opening paragraph on the first page,
which at once brings the reader into the atmosphere of
Anna's environment.
"It was a stormy night in November, and out of doors
the wind swept through the street in a rocking gale, but
in the parlour behind the curio shop life seemed at its ebb.
Old Solny pored over a musty book, and Anna stood with
her head thrown back, her hands clasped behind her, her
eyes seeing dreams; above them on the dun-coloured wall
the Dutch clock ticked methodically, but otherwise the
room was bereft of sound. It was long before either
moved; then Solny stirred automatically?all his actions
were jerky and indirect?and Anna unclasped her hands,
louth is all raw edges to the foibles of age, and Anna
was not yet sixteen. She moved impatiently, and the
dreamy look drifted from her eyes like a shadow
before the sun. 'Father,' she said, 'it's long past eight.'
' So ?' He shut the book reluctantly. ' And the
supper 2 ' ? The supper has been ready for half an
hour.' There was no vexation in her voice; she spoke
with the indifference of one schooled to wait. Life in the
little curio shop, in the little parlour behind it, was one
persistent waiting?for something that never came. The
two sat on in silence while the storm raged. Anna's restless-
ness increased. She broke out abruptly, ' I wish I were a
man ; if I were a man, father, I'd get on board a ship and
be a sailor. At the docks to-day the wind was roaring
through the masts, and it sounded like a weird song; it
made me mad to see the sea. The world and the sea
must be very much alike.' She rested her elbows on the
table and took her face between her hands. Solny
made an unintelligible sound. ' The world is fit for one
thing,' he said?'to keep out of.'" The neighbourhood
of Solny's dwelling was infested with gangs of roughs who
waylaid alien passengers on their way from the vessels.
On this night Anna had heard of a robbery in which a
woman had lost her purse. " She turned again to her father,
' Don't you think the woman who lost her purse was a
fool ? ... If I had been in her place I'd have fought for
what belonged to me?with White or any man.' She tossed
back her plait of red hair and blew some dust from the
cover of a book. ' It must be fine you know, to feel like
that?to feel in the middle of things and not to care.
Here it is just pearls, and china jars, and dust?always.
Don't you ever want to go back 1' ' Back where ?' ' Oh !
back into life; back when you were young.' She paused.
'When I was young,' he said slowly, 'there was persecu-
tion?poverty and persecution?for every Jew, that was
all.' " Suddenly, above the roar of the wind, the sound of a
voice in distress is borne into Anna's strained ears.
" ' Father, did you hear that ?' He turned a page. ' Father I
father! it's a row, I can hear the running and the shouts.
Father!' She clasped her hands. ' Father, I heard some-
one cry out. Give me the lamp.' . . . She flew past him
like a whirlwind and seized the copper lamp. Ten seconds
later he heard her struggling with the bar of the lock.
' Anna,' he called waveringly, ' Anna, think of my
treasures! My stock.' But his only answer was the trail
of smell and smoke that the lamp left."
Then follows a vividly dramatic picture of the scene out-
side, into which Anna had thrown herself ; it is written with
a rare intensity. Every detail of its weirdness stands out
with painful reality. She rushes forward in the direction
from whence the sounds come and meets a wounded,
deformed man, hunted by his pursuers. For the moment he
has got off their track. To rescue him is Anna's first thought.
She hurries him into a place of shelter in a dark passage
adjoining Solny's shop, and then takes him into her father.
' Father, father, are you there? Strike a light 1 . . . The
match flared for an instant, lighting the scene?the shop
with its litter, its cobwebs, its shadows; the bent form of old
Solny, as he held it aloft; the drawn, haggard face of the
rescued man. The united effort verged on the grotesque.
Anna felt herself turn cold again, but she laughed. Real
life is rather terrible, she said huskily, " but?but it's fine
all the same. She sank down suddenly on an empty box.''
The rescued man is taken in and sheltered by Anna and her
father, with the devotion on her part which weakness and
helplessness always excited in her. Her father having, as
an alien, suffered bitterly from persecution, welcomed the
stranger as a fellow sufferer.
His story is before very long drawn reluctantly from
the poor hunted creature. He had been sent over by a
goldsmith in Vienna, for whom he worked, in charge of
jewels, too valuable to be trusted to any delivery than that
of hand; they were to be given direct to the lady for whom
they were intended. He had made friends on the journey
with a man who was a well-known thief, and he, unaware
of his reputation, had on his arrival at the docks, joined him
in lodgings. One night he had taken out the jewels when
alone in the room to polish them, and had been surprised by
a gang of men, who grasped those on the table; he escaped
with the centre jewel of the necklace in the palm of his
hand. He lived in terror of discovery. His master in
Vienna was a hard, cruel man who would demand his pound
of flesh if he discovered the unfortunate Johann. Anna
conceives a plan which, on hearing the story, she puts into
immediate execution; it is that she herself should be the
bearer of the solitary jewel in Johann's possession to the
lady to whom the jewels were addressed. Johann is too
injured and too terrified to venture himself. Anna would
carry them, tell his story, and plead for Johann. She set
out to the West End address given, and finds herself
in the presence of " a woman with a beautiful voice
that did not belong to a beautiful face. A woman
devoid of good looks, but with a personality and a
distinction that were magnetic?a woman who need never
fear a crowd." Mrs. Maxtead's character is a subtle one.
She realised the power of genius and its value. She at once
recognised that Anna was unusual. She became her friend,
provided funds for her education and training as an actress.
And little Anna developed into the great " Solny" before
whom the world bows. We can only regret that space
allows of no further glimpse of her fortunes here.
* " The Circle." By Katherine C. Thurston. (Fublisher:
Blackwood. 1 vol. 6s,)
322 Nursing Section. THE HOSPITAL. March 7, 1903.
jfor 1Reat?ing to tbe Sicf:.
I LOOK TO THEE IN EVERY NEED."
I look to Thee in every need,
And never look in vain ;
I feel Thy touch, Eternal Love,
And all is well again:
The thought of Thee is mightier far
Than sin and pain and sorrow are.
8. longfcllorv.
Every sorrow, every smart,
That the Eternal Father's heart
Hath appointed me of yore,
Or hath yet for me in store,
As my life flows on, I'll take
Calmly, gladly, for His sake.
Anon.
Give free and bold play to those ^instincts of the heart
?which believe that the Creator must care for the creatures
He has made, and that the only real effective care for them
must be that which takes each of them into His love, and
knowing it separately surrounds it with His separate sym-
pathy. There is not one life which the Life-giver ever loses
out of His sight; not one which sins so that He casts it
away; not [one which is not so near to Him that whatever
touches it touches Him with sorrow or with joy.
Phillips Brooks.
What though thy way be dark, and earth
With ceaseless care do cark, till mirth
To thee no sweet strain singeth;
Still hide thy life above, and still
Believe that God is love; fulfil
Whatever lot He bringeth.
Albert E. Evans.
The very least and the very greatest sorrows that God ever
suffers to befall thee, proceed from the depths of His
unspeakable love; and such great love were better for thee
than the highest and best gifts besides that He has given
thee, or ever could give thee, if thou couldst but see it in
this light. So that if your little finger only aches, if you
are cold, if you are hungry or thirsty, if others vex you by
their words or deeds, or whatever happens to you that causes
you distress or pain, it will all help to fit you for a noble and
blessed state.?J. Tauler.
We ought daily or weekly to dedicate a little time to the
reckoning up of the virtues of our belongings?wife, children,
friends?and contemplating them then in a beautiful collec-
tion. And we should do so now, that we may not pardon
and love in vain and too late, after the beloved one has been
taken away from us to a better world.?Jean Paul Richter.
Christ's words assure us that human ties have a living
permanence in Him: that they survive the transitory sphere
in which they have here found their growth; that they
await a resurrection in which they shall be seen in their true
glory,F. WestcotU
?II
IRotes anb ?uerfes.
The Editor is always willing to answer in this column, withoat
any fee, all reasonable questions, as soon as possible.
But the following rules must be carefully observed
I. Every communication must be accompanied by the nam*
and address of the writer.
t. The question must always bear upon nursing, directly ?>
indirectly.
It an answer is required by letter a fee of half-a-crown mutt be
?nclosed with the note containing the inquiry, and we cannot
undertake to forward letters addressed to correspondents making
inquiries. It is therefore requested that our raadera will not
?nclose either a stamp or a stamped envelope.
Middle Ear Deafness.
(166) Will you kindly tell me where I can obtain the treatment
described by Macleod Yearseley. I am troubled with buzzing
tinnitus and paracusis Willisii in my right ear, and, being a nurse,
find it a great drawback in my work ??M. F. B.
Write to Dr. Macleod Yearseley, whose address you will find in
the " Medical Directory."
Glucose.
(167) Can you kindly tell me bow glucose is made for injection
purposes, and also the difference Between "hypodermic and subcu-
taneous injection? "?E. S. E.
A 5 per cent, solution of pure glucose in normal saline solution
is made. Capsules can be obtained of the ttrength required to
make one pint of the solution, when dissolved in boiled water. In-
quire for tnese of your chemist. Hypodermic and subcutaneous
practically mean the same thing.
Operating Room.
(168) I should like to know what is considered the best finish
for the walls of an operating room, whether white-glazed tiles, Kei
cementor, " B opal," or any other material ??L. S.
Opalite is an excellent finish for the purpose. A description of
its practical application is given in an article, " A Pay Hospital
for London," which appeared in The Hospital of January 10.
Patent.
(169) Can anyone tell me whether I can re-patent an article to
which I could add an improvement after the original patentee is
dead ??M. F.
Apply to the Secretary, The Patent Office, 25 Southampton
Buildings, W.C. We believe that you could only patent the im-
provement. But if the improvement were a really important one
its protection would practically protect the original invention for
an extended period, seeing that the one would be useless without
the other.
Medico-Psychological Certificate.
(170) Will you kindly tell me if there are any corrrespondence
classes for those wishing to gain the Medico-Psychological Society's
certificate ? There are no classes held in this asylum.?Irene.
You should endeavour to enter an asylum which prepares its
attendants for this certificate, as both theoretical and practical
instruction is necessary. You will fiod a list of such asylums in
" Burdett's Official Nursing Directory."
Visiting Nurse.
(171) Would you kindly tell me if you think it possible for a
nurse to work up a connection for nursing daily patients with quite
moderate fees. Would you advise advertising, and would such a
venture be most likely to succeed in a large town, or in the country ?
Rita.
We never advise nurses to embark on any enterprise to which
risk is attached, but there is no doubt it could be done with
good introductions. The only way in which such a scheme is
likely to succeed without risk is when it is financed by a committee
of independent persons who can guarantee the nurse "a living wage
whatever her earnings might be.
Important Nursing- Textbooks.
"The Xursing Profession : How and where to Train." 2s. net;
2s. 4d. post free.
"A Handbook for Nurses." (New Edition). 5s.net; 5s. 4d.
post free.
" The Human Body." 5s. post free.
"Ophthalmic Nursing." (New Edition). 3s. Gd. net; 3s. 10d.
post free.
" Gynaecological Nursing." Is. post free.
" Art of Feeding the Invalid." (Popular Edition). Is. 6d. post
free.
" Practical Hints on District Nursing." Is. post free.

				

## Figures and Tables

**Fig. 6. f1:**